# Simulation-Based Multi-Factor Noise-Aware Adaptive Pure Pursuit with Causal EKF-SG Pose Preprocessing for Tracked Agricultural Robots

**DOI:** 10.3390/s26175673

**Published:** 2026-09-07

**Authors:** Fengguo Liu, Liguang Wu, Zhongjun Wu, Gaoshen Cai, Meibao Wang, Shan He

**Affiliations:** 1 School of Mechanical Engineering, Zhejiang Sci-Tech University, Hangzhou 310018, China; 2 Zhejiang Huntsman Intelligent Machinery Co, Ltd., Lishui 323000, China

**Keywords:** pure pursuit, adaptive look-ahead distance, path tracking, agricultural robotics, greenhouse navigation

## Abstract

Accurate and smooth path tracking is important for autonomous tracked agricultural robots operating in greenhouse-like environments. Existing adaptive look-ahead pure-pursuit methods mainly adjust the look-ahead distance according to vehicle speed or path geometry, while the influence of time-varying localization reliability has not been sufficiently considered. This study proposes a noise-aware adaptive pure-pursuit controller that combines Extended Kalman Filter (EKF) estimation with causal Savitzky–Golay (SG) endpoint smoothing. A bounded look-ahead law is designed by jointly considering normalized vehicle speed, lateral error, path curvature, and an innovation-derived localization-noise indicator. Numerical simulations were conducted on straight, circular, S-shaped, and U-shaped reference paths under prescribed localization disturbances. Under the 0.5 m positional-noise condition, the proposed method achieved an root mean square error (RMSE) of 0.087 m and an angular-velocity root mean square (RMS) of 0.28 rad/s, compared with 0.112 m and 0.36 rad/s, respectively, for conventional fixed-look-ahead pure pursuit. Compared with proportional-integral-derivative (PID), Stanley, model predictive control (MPC), and conventional pure-pursuit controllers, the proposed method provides a favorable balance between tracking accuracy and control smoothness. It also has better computational efficiency than MPC while retaining the low-computational-burden advantage of geometric control. In the sensitivity analysis, the relative RMSE increase from 0.1 to 0.8 m was 36.5% for the proposed method and 103.4% for conventional pure pursuit. These results indicate that the proposed lightweight noise-aware control strategy can improve tracking accuracy, control smoothness, and tolerance to localization disturbances under the specified numerical conditions, providing a practical design reference for low-speed greenhouse agricultural robots.

## 1. Introduction

With the continued development of intelligent and automated protected agriculture, the demand for autonomous navigation and precise operation of mobile robots in greenhouses is increasing. Compared with open roads or structured industrial scenarios, greenhouse environments are characterized by narrow passages, frequent changes in path curvature, severe crop occlusion, and strong multipath reflections from metal frames. As a result, tracked mobile robots performing in-row inspection, precision spraying, fruit and vegetable harvesting, and material handling must not only achieve high tracking accuracy, but also maintain control smoothness and chassis stability [1,2,3,4]. Recent review evidence identifies agricultural machinery automation as a practical response to agricultural labor shortages and an enabling framework for precision agriculture and smart-greenhouse operations [5]. For tracked agricultural robots, motion is restricted by nonholonomic constraints, meaning that lateral velocity cannot be generated directly; instead, turning must be achieved through differential coupling of the two tracks [6,7]. Therefore, path-tracking controllers must exhibit strong adaptability to complex path geometry, environmental sensing noise, and the nonlinear dynamic response of the chassis.

As one of the most classical and widely used geometric control methods for mobile robot path tracking, pure pursuit has been extensively applied to low-speed platforms and agricultural vehicle navigation because of its simple principle, clear geometric interpretation, low computational burden, and strong real-time performance [8]. However, the traditional pure-pursuit algorithm typically uses a fixed look-ahead distance, which cannot satisfy the precise tracking requirements in complex greenhouse environments and on paths with varying curvature [9,10,11].

To address the limitations of fixed-look-ahead pure pursuit, existing studies have explored several alternative and adaptive path-tracking strategies. However, these approaches involve different practical trade-offs: Stanley-based controllers use lateral and heading errors to improve geometric correction, yet noisy error signals, sideslip, and increasing operating speed can lead to oscillatory steering [12,13,14]. PID controllers are simple and easy to implement, but their derivative action can amplify high-frequency measurement noise, resulting in overshoot and jitter when tracking complex paths [15,16]. MPC can incorporate preview information and explicitly handle state and control constraints, but its online optimization increases computational and tuning requirements for embedded agricultural platforms [17,18,19,20]. Adaptive pure-pursuit methods adjust the look-ahead distance according to vehicle speed, path curvature, or other motion-related variables, thereby reducing part of the responsiveness–smoothness trade-off [21,22,23,24]. However, the direct influence of localization reliability on look-ahead regulation remains insufficiently characterized in the tracked greenhouse-like setting considered here. This study therefore focuses on integrating pose preprocessing and an innovation-derived localization-noise indicator into a bounded adaptive pure-pursuit framework.

Based on the above considerations, this study investigates a multi-factor, noise-aware adaptive pure-pursuit controller for a tracked robot on greenhouse-like reference paths. Within the scope of a simulation-based feasibility assessment, the main contributions are as follows:

1. A pose-feedback preprocessing strategy combining EKF estimation and causal Savitzky–Golay endpoint smoothing is introduced to improve the continuity of localization feedback before it enters the path-tracking controller.
2. A bounded noise-aware adaptive look-ahead distance law is developed by jointly considering vehicle speed, lateral error, path curvature, and localization-noise level, which distinguishes the proposed method from adaptive pure-pursuit methods mainly based on speed or curvature.
3. A Lyapunov-based stability analysis is provided for the kinematic tracking controller under stated assumptions, offering a theoretical basis for the controller design.
4. Numerical simulations are conducted on straight, circular, S-shaped, and U-shaped greenhouse-like reference paths under prescribed localization disturbances, and the proposed method is compared with PID, Stanley, MPC, and conventional fixed-look-ahead pure pursuit in terms of tracking accuracy, angular-velocity smoothness, and computational cost.

## 2. Method

### 2.1. Localization Signal Source and Noise Characterization

Path tracking of a tracked mobile robot requires continuous position and heading feedback. The robot pose at the sampling instant k is defined as

(1) $$P_{k}=[x_{k},y_{k},\theta_{k}{]}^{T}$$

where $x_{k}$ and $y_{k}$ denote the global position coordinates and $\theta_{k}$ is the heading angle.

To evaluate the robustness of the proposed controller under localization disturbances, the observation model is expressed as

(2) $$z_{k}^{p}=p_{k}+\nu_{k}+b_{k},$$

where $\nu_{k}$ denotes measurement noise and $b_{k}$ represents intermittent disturbances in the localization signal. This formulation separates the nominal pose from the random and abrupt components of the observation. The observation is first processed by the EKF and then by the causal SG smoother before being used for adaptive look-ahead-distance calculation and pure-pursuit control.

### 2.2. Noise-Robust Pose Estimation Framework

To improve the reliability of pose feedback in greenhouse environments characterized by dense crop occlusion, frequent metal-frame reflections, multipath interference, and complex variable-curvature paths, a noise-robust pose estimation framework combining an EKF and SG smoothing is adopted. The framework is designed to suppress random measurement noise and intermittent non-line-of-sight (NLOS)-induced disturbances while preserving the continuity of the robot path. In this way, the filtered pose output can serve as a more stable input to the subsequent adaptive pure-pursuit controller. The processing flow can be summarized as: localization observation → EKF prediction and correction → causal SG smoothing → adaptive pure pursuit.

Considering the motion characteristics of tracked agricultural robots, the system state is defined as

(3) $$x_{k}={\left[\begin{array}{cccc} x_{k} & y_{k} & v_{k} & \theta_{k} \end{array}\right]}^{T}$$

where $x_{k}$ and $y_{k}$ are the robot’s position coordinates in the global frame, *v_k_* is the linear speed, and $\theta_{k}$ is the heading angle.

With sampling period Δt, acceleration input $a_{k}$, and angular-velocity input $\omega_{k}$, the discrete state-transition model is

(4) $$x_{k+1}=f(x_{k},u_{k})+w_{k}=\left[\begin{array}{c} x_{k}+v_{k}\cos\theta_{k}\Delta t\\ y_{k}+v_{k}\sin\theta_{k}\Delta t\\ v_{k}+a_{k}\Delta t\\ \theta_{k}+\omega_{k}\Delta t \end{array}\right]+w_{k}$$

where $x_{k+1}$ is the state at the next sampling instant, f(⋅) is the nonlinear state-transition function, $u_{k}=[a_{k},\omega_{k}{]}^{T}$ is the control-input vector, Δt is the sampling period, $a_{k}$ is the longitudinal acceleration, and $\omega_{k}$ is the angular velocity, and $w_{k}$ is the process-noise vector. For the constant-speed simulations, $a_{k}=0$. The heading angle is wrapped to the interval (−π,π].

The observation model is

(5) $$z_{k}^{p}=Hx_{k}+\nu_{k}+b_{k},\;H=\left[\begin{array}{cccc} 1 & 0 & 0 & 0\\ 0 & 1 & 0 & 0\\ 0 & 0 & 0 & 1 \end{array}\right]$$

where $z_{k}^{p}$ is the measured position-and-heading vector, H is the observation matrix that extracts $x_{k},y_{k},\;and\;\theta_{k}$ from the state, $\nu_{k}$ is measurement noise, and $b_{k}$ is an intermittent localization disturbance.

The EKF prediction step is calculated as

(6) $$\begin{array}{rr} {\hat{x}}_{k|k-1} & =f({\hat{x}}_{k-1|k-1},u_{k-1})\\ \Sigma_{k|k-1} & =F_{k-1}\Sigma_{k-1|k-1}F_{k-1}^{T}+Q_{k-1} \end{array}$$

where ${\hat{x}}_{k|k-1}$ is the predicted state, ${\hat{x}}_{k-1|k-1}$ is the posterior state estimate at the previous sampling instant, $\Sigma_{k|k-1}$ is the predicted state covariance, $\Sigma_{k|k-1}$ is the previous posterior covariance, $F_{k-1}$ is the Jacobian matrix of the state-transition function, and $Q_{k-1}$ is the process-noise covariance matrix. The notation k|k−1 denotes a prior estimate based on measurements up to k−1.

The EKF correction step is

(7) $$\begin{array}{rr} \eta_{k} & =z_{k}^{p}-H{\hat{x}}_{k|k-1}\\ S_{k} & =H\Sigma_{k|k-1}H^{T}+R_{k}\\ K_{k} & =\Sigma_{k|k-1}H^{T}S_{k}^{-1}\\ {\hat{x}}_{k|k} & ={\hat{x}}_{k|k-1}+K_{k}\eta_{k}\\ \Sigma_{k|k} & =(I-K_{k}H)\Sigma_{k|k-1} \end{array}$$

where $\eta_{k}$ is the measurement innovation, $S_{k}$ is the innovation covariance, $K_{k}$ is the Kalman gain, $R_{k}$ is the measurement-noise covariance, ${\hat{x}}_{k|k}$ is the posterior state estimate, $\Sigma_{k|k}$ is the posterior state covariance, and I is the identity matrix. The superscript T denotes matrix transpose, and the superscript −1 denotes matrix inversion. The heading component of $\eta_{k}$ is wrapped to (−π,π].

The EKF posterior pose is then processed by a causal 11-sample, third-order SG endpoint smoother:

(8) $${\tilde{q}}_{k}=\sum_{i=0}^{10}h_{i}{\hat{q}}_{k-10+i}$$

where $q_{k}$ denotes one pose component, such as $x_{k},\;y_{k}$, or $\theta_{k}$; ${\hat{q}}_{k-10+i}$ is the corresponding EKF posterior estimate; $q_{k}$ is the SG-smoothed value; $h_{i}$ is the i-th SG filter coefficient; and i = 0, …, 10 indexes the current sample and ten previous samples. Because only current and previous samples are used, the SG filter is causal and does not require future measurements. The innovation $\eta_{k}$ and its covariance $S_{k}$ are calculated during the EKF correction step and retained for subsequent localization-quality assessment. Meanwhile, the SG-smoothed pose is used to determine the nearest path point and the corresponding tracking error. These two outputs provide the necessary inputs for the adaptive look-ahead-distance calculation described below.

### 2.3. Multi-Factor Dynamic Adaptive Look-Ahead Distance Design

To overcome the limitations of fixed look-ahead distance under varying speed, curvature, and localization noise, a multi-factor adaptive look-ahead distance strategy is developed by jointly considering the robot motion state, path geometry, tracking condition, and localization quality [25].

The speed, lateral-error, and curvature terms are first normalized as

(9) $$\begin{array}{rr} {\bar{v}}_{k} & =clip\left(\frac{v_{k}}{v_{ref}},0,1\right)\\ {\bar{e}}_{k} & =clip\left(\frac{\left|e_{k}\right|}{e_{ref}},0,1\right)\\ {\bar{\kappa }}_{k} & =clip\left(\frac{\left|\kappa_{k}\right|}{\kappa_{ref}},0,1\right) \end{array}$$

where ${\bar{v}}_{k}$, ${\bar{e}}_{k}$, and ${\bar{\kappa }}_{k}$ are dimensionless normalized variables. $v_{k}$ is the robot linear speed in m/s, $v_{ref}$ is the reference speed, $e_{k}$ is the signed lateral tracking error in m, and $e_{ref}$ is the reference lateral error. $\kappa_{k}$ is the local reference-path curvature in $m^{-1}$, and $\kappa_{ref}$ is the reference curvature. The function clip(z,0,1) limits the input z to the interval [0,1]. For the numerical simulations reported here, $v_{ref}=0.8\;m/s$, $e_{ref}=0.6\;m$, and $\kappa_{ref}=0.35\;m^{-1}$.

The localization-quality indicator is calculated from the EKF innovation before SG smoothing:

(10) $$d_{k}=\eta_{k}^{T}S_{k}^{-1}\eta_{k},\;\rho_{k}=0.92\rho_{k-1}+0.08clip\left(\frac{\sqrt{d_{k}/3}}{2},0,1\right),\;\rho_{0}=0$$

where $\eta_{k}$ and $S_{k}$ are the EKF innovation and its covariance obtained from the correction equations above. $d_{k}$ is the normalized innovation squared and is dimensionless. The value 3 denotes the dimension of the position-and-heading observation. $\rho_{k}$ is the dimensionless localization-noise indicator, 0.92 is the low-pass memory coefficient, 0.08 is the update coefficient, and $\rho_{0}$ is the initial noise indicator.

The adaptive look-ahead distance is then calculated as

(11) $$l_{d,k}=clip\left[l_{0}(1+a_{v}{\bar{v}}_{k}-a_{e}{\bar{e}}_{k}-a_{\kappa }{\bar{\kappa }}_{k}+a_{\rho }\rho_{k}),l_{min},l_{max}\right]$$

where $l_{d,k}$ is the adaptive look-ahead distance, $l_{0}$ is the base look-ahead distance, and $l_{min}$ and $l_{max}$ are its lower and upper bounds. The coefficients $a_{v}$, $a_{e}$, $a_{\kappa }$, and $a_{\rho }$ are dimensionless weighting coefficients for normalized speed, lateral error, path curvature, and localization noise, respectively. Therefore, the term inside the square brackets is dimensionless, and $l_{d,k}$, $l_{0}$, $l_{min}$, and $l_{max}$ have units of meters. The function clip(⋅) limits the calculated distance to the interval $\left[l_{min},l_{max}\right]$.

The speed and localization-noise terms increase the look-ahead distance. The speed term improves path anticipation, while the localization-noise term reduces the sensitivity of the controller to short-term pose fluctuations. In contrast, the lateral-error and curvature terms reduce the look-ahead distance to improve local error correction and turning responsiveness. The lower and upper bounds prevent excessive steering sensitivity and excessive tracking delay, respectively.

The weighting coefficients and look-ahead bounds were selected using a staged heuristic tuning procedure. First, $l_{0}$, $l_{min}$, and $l_{max}$ were determined from the nominal operating speed, vehicle size, turning capability, target-point range, and actuator limitations. The speed coefficient $a_{v}$ was then adjusted using the straight-path condition to balance preview capability and steering smoothness. The lateral-error coefficient $a_{e}$ and curvature coefficient $a_{\kappa }$ were tuned using the circular, S-shaped, and U-shaped paths to reduce lateral deviation and corner cutting while avoiding excessive steering oscillation. Finally, the localization-noise coefficient $a_{\rho }$ was adjusted under controlled measurement disturbances so that the look-ahead distance increased during unreliable pose feedback without causing excessive tracking delay.

The parameters were evaluated using the lateral root-mean-square error (RMSE), maximum absolute lateral error, and root-mean-square angular velocity, together with the requirements that the look-ahead distance and control commands remain bounded. The numerical simulations reported here are $l_{0}=1.4\;m$, $l_{min}=0.8\;m$, $l_{max}=2.4\;m$, $a_{v}=0.45$, $a_{e}=0.80$, $a_{\kappa }=0.80$, and $a_{\rho }=0.35$.

Once $l_{d,k}$ has been determined, it is passed to the subsequent pure-pursuit control law. The smoothed pose is used to determine the target point and heading error, while $l_{d,k}$ determines the geometric curvature command.

### 2.4. Adaptive Pure-Pursuit Control Law and Stability Analysis

**Figure 1:**
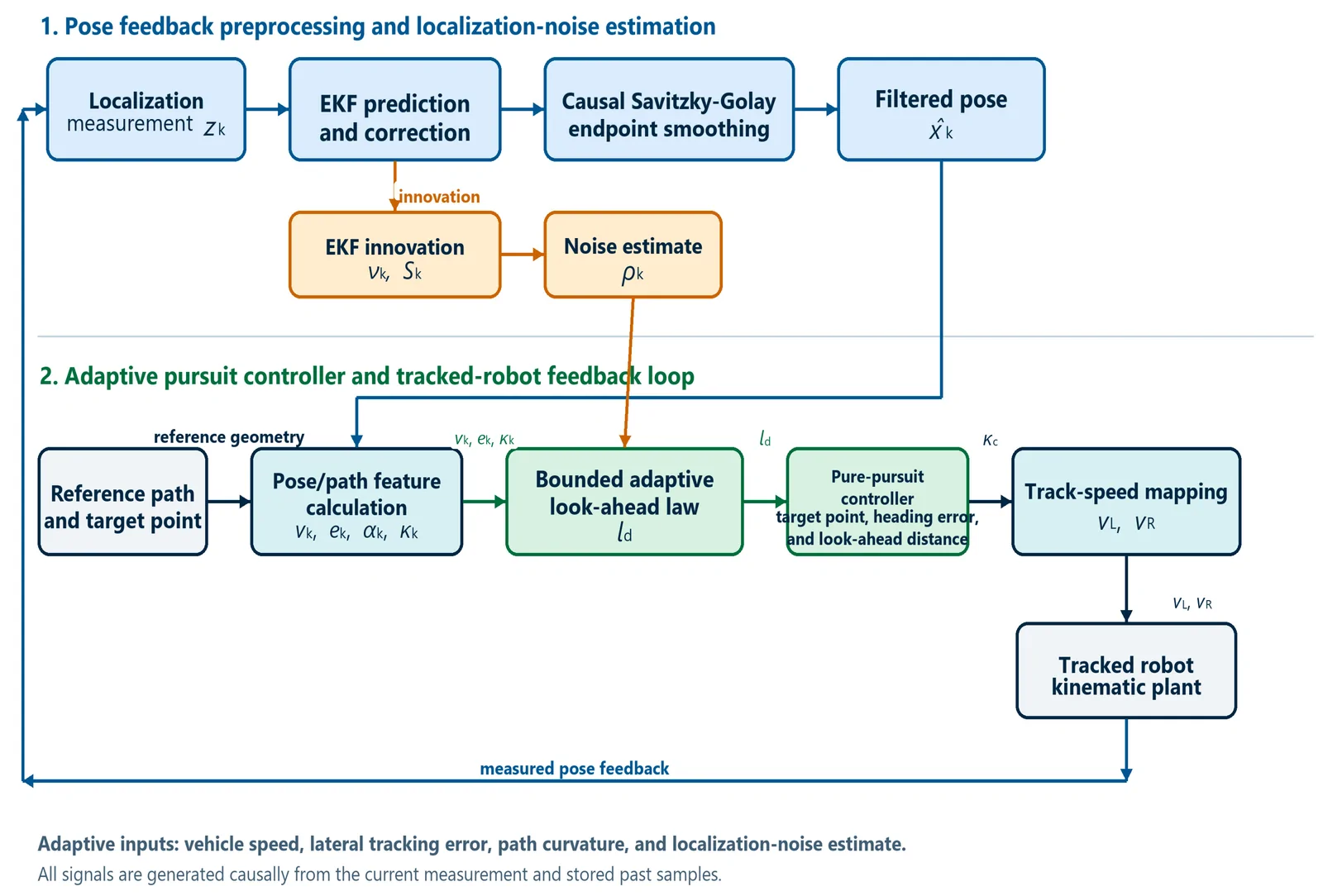
Closed-loop framework of the proposed noise-aware adaptive pure-pursuit controller.

The filtered pose, localization-noise estimate, adaptive look-ahead law, and pure-pursuit controller are organized into the closed-loop framework shown in Figure 1. The resulting curvature command is converted into left-track and right-track velocity commands and applied to the tracked-robot kinematic plant, whose measured pose is fed back to the localization input.

For the current filtered robot pose and the target point selected from the reference path, the pure-pursuit law assumes that the robot follows a circular arc connecting its current position to the target point [26]. The corresponding curvature command is given by

(12) $$\kappa_{c}=\frac{2\sin\alpha }{l_{d}}$$

where α is the heading error between the robot orientation and the direction of the target point, and $l_{d}$ is the adaptive look-ahead distance. The angular velocity command is then generated according to the current linear velocity and the desired curvature.

For the tracked differential-drive platform, the computed curvature command is converted into left and right track velocity commands and executed by the vehicle motion controller. To ensure practical operation, all control outputs are constrained within the physical limits of the drive system.

To evaluate the stability of the proposed controller, the lateral tracking error e and heading error α are considered as the system states. A Lyapunov candidate function is selected as

(13) $$V=\frac{1}{2}e^{2}+\lambda (1-\cos\alpha )$$

Among them, λ>0 is a positive constant. The function is positive definite and reaches zero only when both tracking errors vanish [27].

Under the assumptions that the reference path curvature is bounded, the vehicle speed remains within a finite range, and the adaptive look-ahead distance is constrained between predefined lower and upper bounds, the closed-loop system can be regarded as a bounded time-varying system. The adaptive pure-pursuit law introduces negative feedback on both lateral and heading errors, ensuring that the Lyapunov function remains non-increasing during system evolution. According to Lyapunov stability theory and the LaSalle invariance principle, the tracking errors remain bounded and gradually converge toward the equilibrium point.

Therefore, the proposed adaptive pure-pursuit controller guarantees stable path tracking while preserving the simplicity and real-time characteristics of the classical pure-pursuit algorithm. By integrating the adaptive look-ahead distance mechanism, the controller achieves a better balance between tracking accuracy, control smoothness, and robustness to localization uncertainty, making it suitable for greenhouse navigation of tracked agricultural robots.

## 3. Simulation Experiments and Results

### 3.1. Simulation Platform and Parameter Settings

To verify the effectiveness of the proposed multi-factor dynamic adaptive look-ahead pure-pursuit control method for path tracking of tracked Automated Guided Vehicles (AGVs) in agricultural greenhouses, a closed-loop path-tracking control simulation system was established on the MATLAB R2021b/Simulink platform [28]. The system mainly includes a localization signal output module, an EKF–SG pose preprocessing module, a dynamic look-ahead distance adjustment module, and an adaptive pure-pursuit control module, and can simulate the path-tracking process of a tracked robot in a greenhouse environment under noise interference. The controller is implemented in discrete time with a sampling period of 0.02 s. The chassis is modeled using a differential-drive tracked kinematic model, and the linear speed is set to 0.5 m/s. The angular-velocity command is limited to 1.2 rad/s. The nominal vehicle geometry and track spacing are defined according to the tracked-robot model used in the simulation.

Considering typical greenhouse operation scenarios, four types of paths are selected for simulation: straight, circular, S-shaped, and U-shaped paths. To ensure consistency between the mathematical definitions and the plotted trajectories, the reference paths were defined as follows.

The straight path was defined as

(14) $$p_{st}(s)=\left[\begin{array}{c} x_{st}(s)\\ y_{st}(s) \end{array}\right]=\left[\begin{array}{c} s\\ 3 \end{array}\right],\;1\leq s\leq 9$$

where $p_{st}(s)$ denotes the reference position, $x_{st}$ and $y_{st}$ are the reference coordinates in meters, and s is the longitudinal path parameter in meters.

The circular path was defined as

(15) $$p_{c}(\theta )=\left[\begin{array}{c} x_{c}(\theta )\\ y_{c}(\theta ) \end{array}\right]=\left[\begin{array}{c} 5+4\cos\theta \\ 3+4\sin\theta \end{array}\right],\;0\leq \theta \leq 2\pi$$

where $p_{c}(\theta )$ denotes the circular reference position, θ is the angular path parameter in radians.

The S-shaped path was defined as

(16) $$p_{S}(s)=\left[\begin{array}{c} x_{S}(s)\\ y_{S}(s) \end{array}\right]=\left[\begin{array}{c} s\\ 3+2\sin(1.2s)+0.5s \end{array}\right],\;1\leq s\leq 9$$

where $p_{S}(s)$ denotes the S-shaped reference position, s is the longitudinal path parameter in meters, and 0.7 m is the lateral amplitude of the sinusoidal path.

The U-shaped path consisted of two straight segments connected by a semicircular segment. The three segments were defined as

(17) $$p_{U,1}(u)=\left[\begin{array}{c} u\\ 2 \end{array}\right],\;1\leq u\leq 9\;p_{U,2}(\theta )=\left[\begin{array}{c} 5+4\cos\theta \\ 2+4\sin\theta \end{array}\right],\;0\leq \theta \leq \pi \;p_{U,3}(u)=\left[\begin{array}{c} u\\ 6 \end{array}\right],\;1\leq u\leq 9$$

where $p_{U,1}$ is the first straight segment, u is its local path parameter in meters, $p_{U,2}$ is the semicircular segment, θ is the angular parameter in radians, $p_{U,3}$ is the second straight segment and u is its local path parameter in meters. The straight segments are located at y = 2 m and y = 6 m, while the semicircular segment has a center at (5,2) m and a radius of 4 m. The reference heading and curvature used by the controllers were calculated from the first-order and second-order derivatives of the corresponding trajectories. These paths are idealized numerical benchmarks for evaluating different geometric characteristics and do not represent measured greenhouse trajectories.

The simulations use prescribed positional-disturbance levels rather than field-calibrated greenhouse measurements. The positional-noise standard deviation for the main comparison is 0.5 m. Additional sensitivity conditions use positional-noise standard deviations of 0.1 m, 0.3 m, 0.5 m, and 0.8 m. Position disturbances are applied independently to the x-coordinates and y-coordinates. The heading-measurement noise standard deviation is $2^{\circ }$ (0.0349 rad), the positional impulse-disturbance standard deviation is 0.12 m, and the heading impulse-disturbance standard deviation is 4 degrees, with an impulse occurrence probability of 0.02 at each sampling instant. The positional-noise levels must not be interpreted as heading-noise levels.

The process-noise covariance matrix Q represents uncertainty in the tracked-robot motion model, including slight slip and unmodeled disturbances. The observation-noise covariance matrix R represents measurement uncertainty in the position coordinates and heading angle. The position and heading components of R are selected according to $\sigma_{p}$ and $\sigma_{\theta }$, respectively, so that the covariance settings are consistent with the separately specified position and heading noise levels.

In the pose preprocessing stage, EKF and SG smoothing are combined to improve the pose signal quality fed into the controller. The EKF first performs model-based state prediction and measurement correction. The EKF output sequence is then passed to a causal SG endpoint-smoothing module with a filter window length of 11 and a polynomial order of 3. The endpoint estimate uses the current EKF output and the ten previous outputs; therefore, future samples are not required. During initialization, the available history is used until a complete 11 sample window is formed. This causal filtering process reduces residual high-frequency fluctuations while preserving local path trends and providing smoother state feedback for the subsequent adaptive look-ahead controller.

The paths and disturbance levels used in the simulations are idealized numerical test conditions rather than direct measurements of a specific greenhouse. The 0.5 m level is used for the main performance comparison, while 0.1 m, 0.3 m, 0.5 m, and 0.8 m are used as prescribed sensitivity-analysis levels. These values do not establish the statistical distribution or practical frequency of localization errors in real greenhouse operation. Actual errors may be non-Gaussian, temporally correlated, state-dependent, and influenced by crop occlusion, metal-frame reflections, multipath propagation, and robot motion.

### 3.2. Typical Path-Tracking Results

To verify the adaptability of the proposed multi-factor dynamic adaptive look-ahead pure-pursuit control method under different path geometries, simulations are conducted on straight, circular, S-shaped, and U-shaped agricultural operation trajectories. The proposed method is compared with conventional fixed-look-ahead pure pursuit, Stanley, PID, and MPC. Qualitative analysis is carried out through path-tracking curves, lateral error curves, adaptive look-ahead distance curves, and pose response curves before and after filtering.

**Figure 2:**
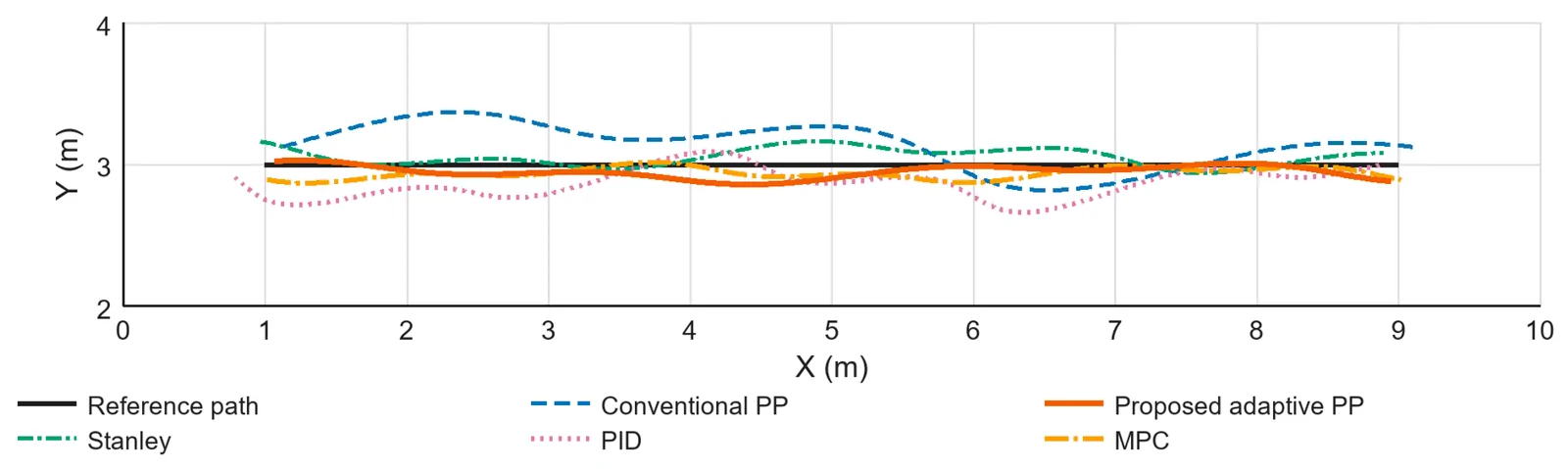
Comparison of trajectory tracking results of different control algorithms (proposed adaptive pure pursuit (PP), conventional PP, Stanley, PID, and MPC) under straight-path conditions.

**Figure 3:**
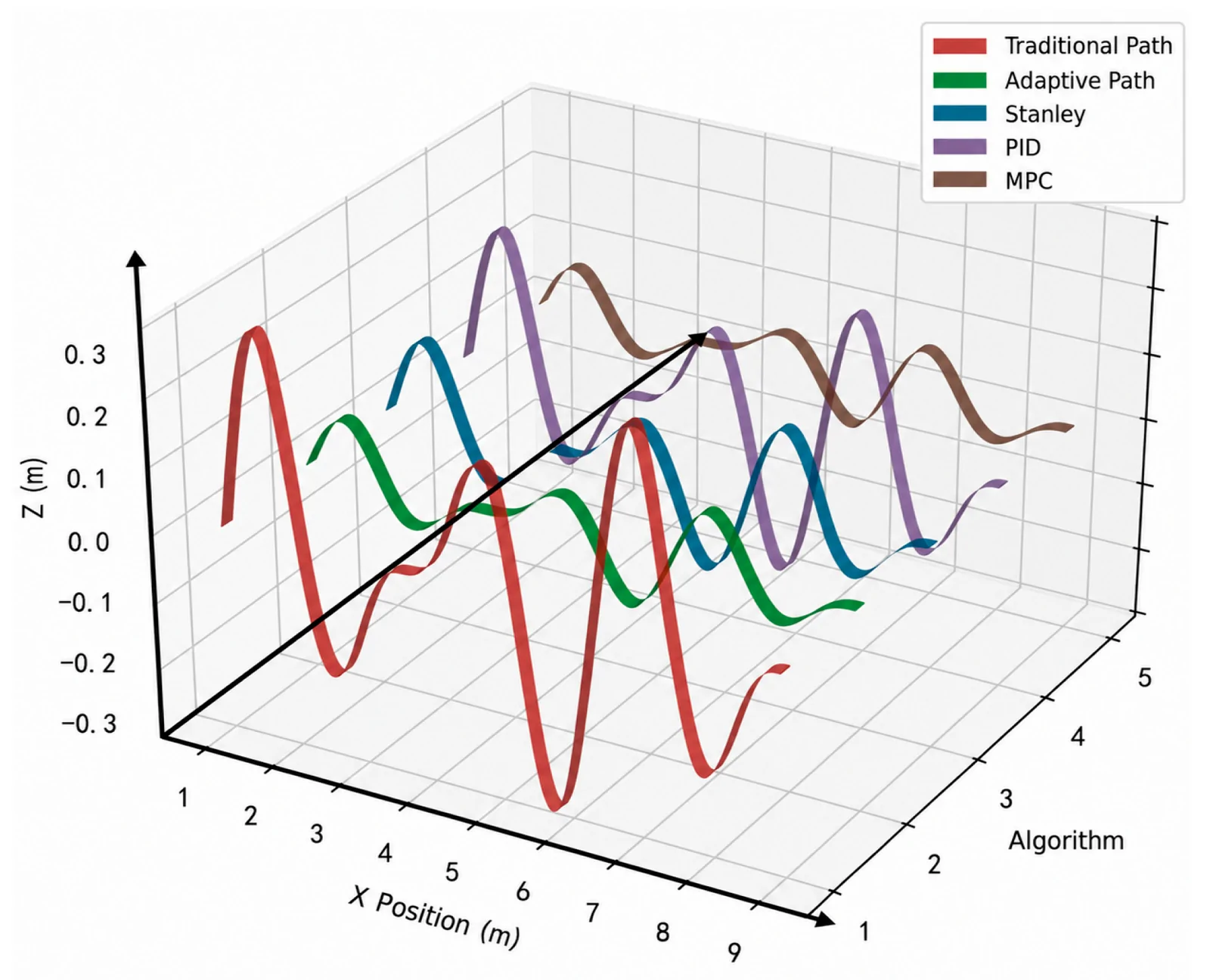
Quantitative comparison of lateral tracking error distributions for different path-tracking algorithms under straight-line trajectory conditions.

On a straight path, the curvature of the reference path is close to zero, and the path changes are gentle, mainly used to test the stability of the system and the ability to maintain a straight line under low curvature conditions. As shown in Figure 2, the simulation results indicate that even if there is an initial deviation, the proposed method can quickly guide the robot to return to a position close to the reference path and maintain a small lateral error fluctuation. Due to the combined effect of the speed term and the error term, the predicted distance on the straight segment remains relatively stable, resulting in a smaller angular velocity change and a smooth overall path with no obvious high-frequency oscillations. To further quantitatively compare the tracking accuracy and error distribution of different path-tracking controllers, in the linear-path simulation condition, the lateral offset distance along the equally spaced sampled path is taken, and the obtained error time series is plotted in Figure 3 to reflect the stability of the tracking and the offset amplitude. In contrast, the traditional fixed look ahead pure-pursuit method can also achieve path convergence, but it still shows slight angular velocity fluctuations under positioning noise; Stanley converges faster on the straight segment, but is more sensitive to control input; PID and MPC can maintain basic tracking, but their smoothness and response continuity are not as good as the proposed method.

**Figure 4:**
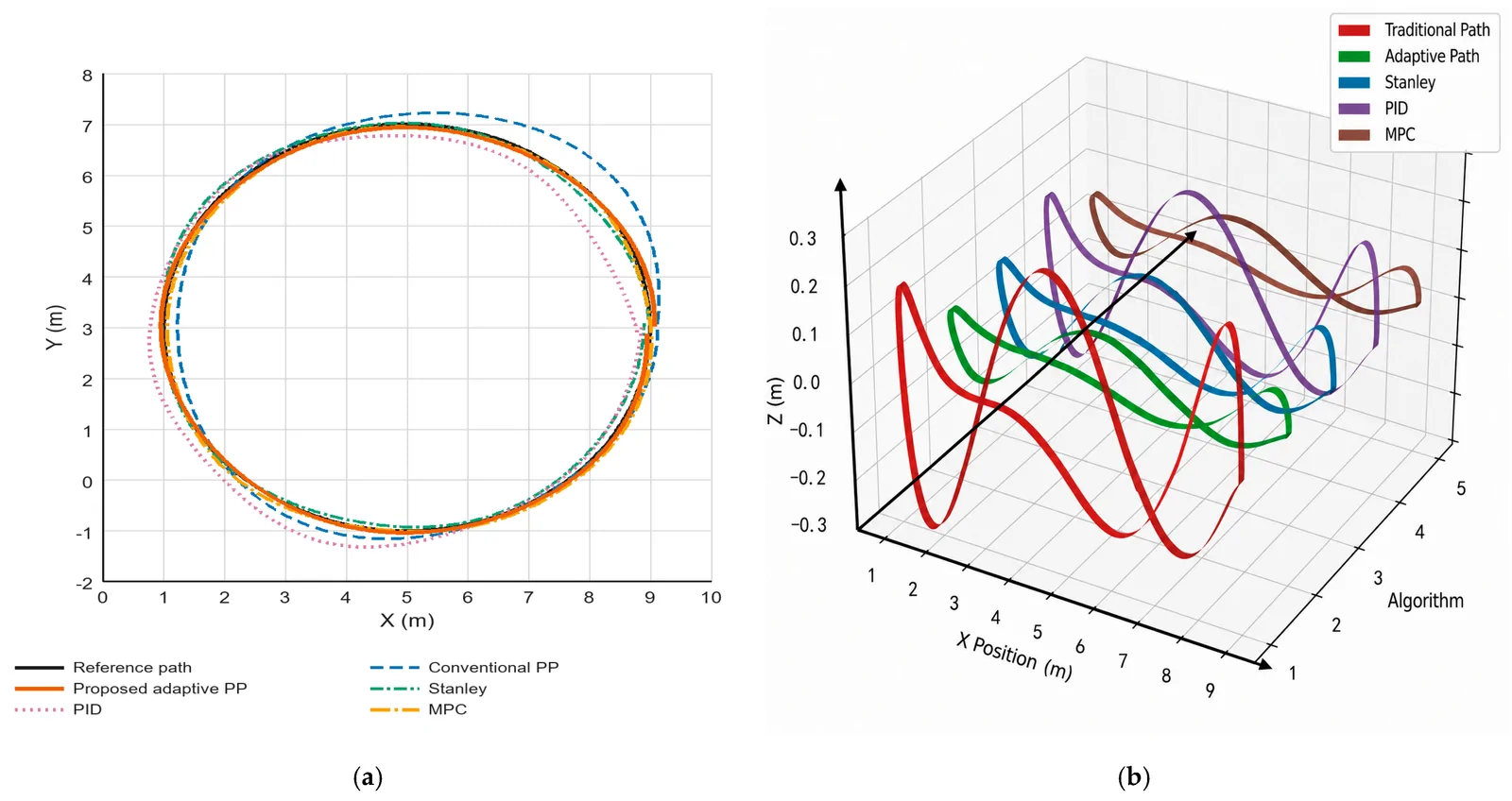
Tracking performance on circular path. (**a**) trajectory comparison of different controllers, (**b**) corresponding lateral tracking error curves.

On the circular path, as shown in Figure 4, the curvature remains constant, and the turning radius is small, which imposes high requirements on sustained steering capability. For fixed-look-ahead pure pursuit, a larger look-ahead distance causes outward deviation after entering the arc segment and reduces path adherence; a smaller look-ahead distance makes the control output more responsive, but it is also more prone to local oscillations under noise. The proposed method automatically reduces the look-ahead distance on circular paths according to local curvature, thereby increasing local sensitivity in target-point search and helping the robot enter a stable turning state faster. Meanwhile, the EKF–SG-preprocessed pose signal is smoother, and the angular velocity output is continuous without obvious spikes, indicating good geometric adaptability and control smoothness on constant-curvature paths. By comparison, Stanley converges faster on the circular arc but shows some oscillation in high-curvature transition regions; PID is prone to steady-state bias in sustained turning; and MPC has strong path-fitting ability but higher computational cost.

**Figure 5:**
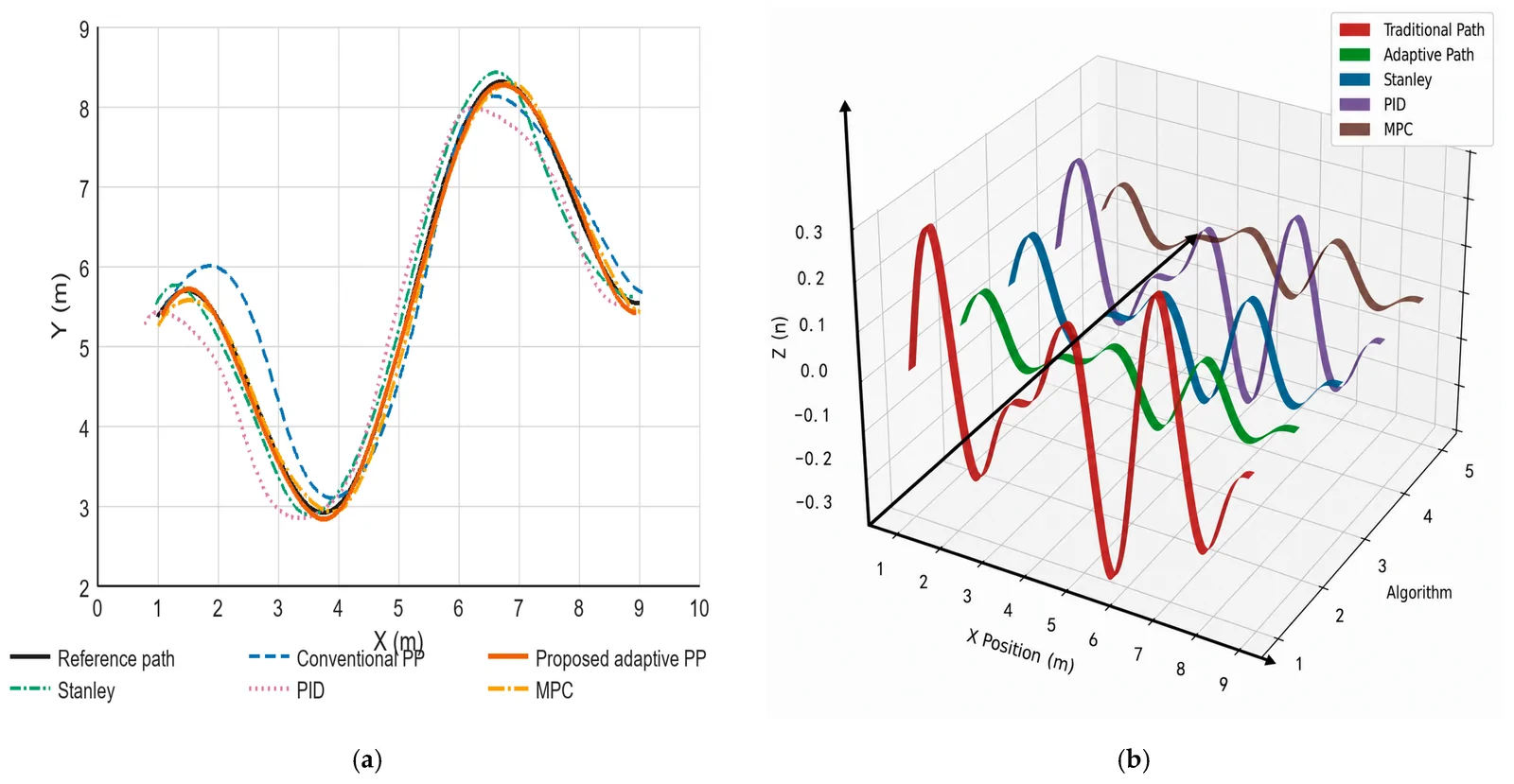
Tracking performance on S-shaped path with varying curvature. (**a**) trajectory tracking results, (**b**) lateral tracking error response of different control methods.

On the S-shaped path, as shown in Figure 5, curvature continuously changes with position, making it a key scenario for testing dynamic adaptability. The simulation results show that conventional fixed-look-ahead pure pursuit tends to exhibit ‘delay when entering the turn and overshoot when leaving the turn,’ and the lateral error curve fluctuates significantly in curvature transition segments. This is fundamentally because a fixed look-ahead distance cannot simultaneously satisfy the control requirements of the straight section and the turning section. By fusing path curvature, lateral error, and real-time speed, the proposed method dynamically adjusts the look-ahead distance: it shortens the look-ahead distance before entering a curve, improving responsiveness to path changes; after leaving the curve, the look-ahead distance gradually increases as curvature decreases, thereby suppressing excessive correction. As a result, the robot maintains good continuous tracking on the S-shaped path, and the angular velocity curve becomes smoother. Compared with conventional methods, the proposed method does not show obvious oscillation at curvature switching points, demonstrating the clear advantage of dynamic look-ahead distance on continuously varying-curvature paths.

**Figure 6:**
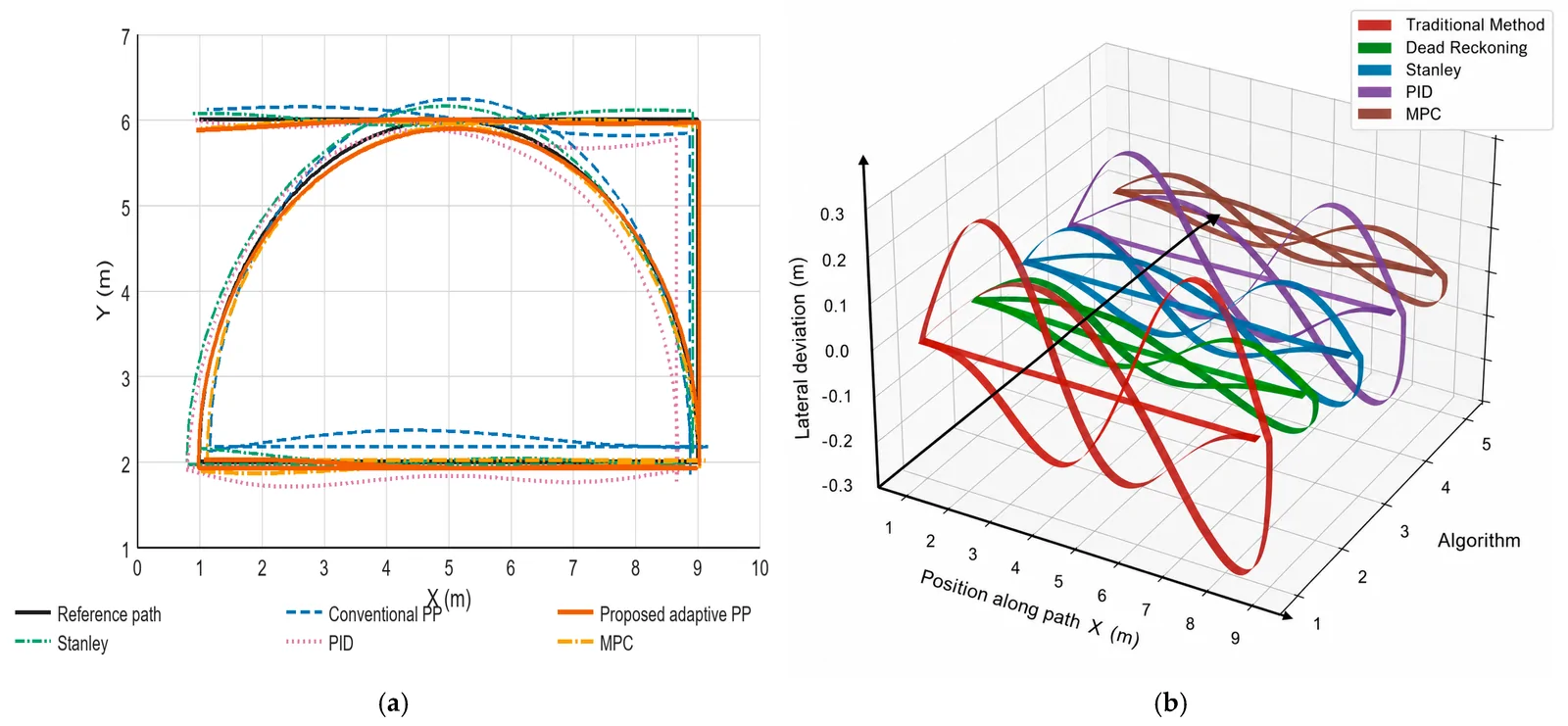
Tracking performance on U-shaped path: (**a**) trajectory comparison of different controllers during large-angle turning, (**b**) corresponding lateral tracking error curves.

On the U-shaped path, as shown in Figure 6, the robot must complete a large-angle U-turn, which places higher demands on steering sensitivity, error convergence, and stability. The simulation results show that fixed-look-ahead pure pursuit tends to expand outward noticeably on the U-shaped path, especially in the transition regions when entering and leaving the curve, because target-point selection becomes sluggish and path deviation increases. Stanley can correct the heading quickly, but its angular velocity changes substantially, which can induce strong control fluctuations in the U-turn segment. PID has relatively insufficient response in large-curvature segments, leading to slower path convergence. In contrast, the proposed method automatically shortens the look-ahead distance according to path curvature and lateral error, forming a more compact tracking path in the U-turn segment and restoring a larger look-ahead distance in time after leaving the curve, thereby avoiding excessive steering and path oscillation. This result indicates that the dynamic adaptive look-ahead strategy has stronger geometric adaptability and control stability on large-curvature U-turn paths.

From the overall pose response perspective, the combined EKF and SG preprocessing significantly improves the quality of the input signal to the controller. Raw localization data with Gaussian noise and NLOS abrupt disturbances introduce short-term jumps in the path, whereas the preprocessed pose output is more continuous and the high-frequency fluctuations in the lateral error curve are significantly reduced. The difference between the trajectories before and after filtering is particularly evident in turning segments, and the preprocessed control input reflects the true motion state more accurately, reducing noise interference in look-ahead distance calculation and angular velocity generation. This shows that pose preprocessing not only improves localization accuracy but also directly improves control stability and smoothness.

The qualitative results across the four paths show that the proposed method maintains good smoothness on straight paths, exhibits strong curvature adaptability on circular paths, demonstrates strong continuous variable-curvature tracking on S-shaped paths, and provides better large-angle U-turn control on U-shaped paths. Overall, the dynamic look-ahead distance mechanism combined with EKF–SG preprocessing effectively alleviates the lag, overshoot, and chattering problems of conventional fixed-look-ahead pure pursuit in complex agricultural paths, providing intuitive support for the subsequent quantitative analysis.

## 4. Performance Evaluation and Robustness Validation

To further quantitatively evaluate the proposed multi-factor dynamic adaptive look-ahead pure-pursuit control method, a comprehensive analysis is carried out from the perspectives of tracking accuracy, control smoothness, real-time computation capability, and noise robustness. All comparison algorithms use the same reference path, prescribed disturbance condition, sampling period, initial condition, and actuator constraint. The reported values are retained single-run numerical results and are presented descriptively; no repeated-trial statistics are claimed.

### 4.1. Comparison of Tracking Performance

To comprehensively evaluate the performance of the proposed method, conventional fixed-look-ahead pure pursuit, Stanley, PID, and MPC are selected as comparison algorithms. The fixed-look-ahead pure pursuit serves as a baseline geometric controller; Stanley is used to compare lateral-error-driven control; PID tests the adaptability of classical feedback control in agricultural path tracking; and MPC is used to assess the trade-off between tracking accuracy and computational burden [29]. All algorithms are simulated under the same path, noise conditions, and sampling period to ensure fair comparison.

Table 1 summarizes the available performance values under the prescribed 0.5 m positional-noise standard deviation. RMSE evaluates overall path-tracking accuracy, maximum lateral error represents the largest lateral deviation, RMS angular velocity reflects control smoothness, and computation time indicates computational burden. The proposed method reports an RMSE of 0.087 m and a maximum lateral error of 0.198 m, compared with 0.112 m and 0.267 m for conventional fixed-look-ahead pure pursuit.

For the same prescribed 0.5 m positional-noise condition, the retained angular-velocity RMS is 0.28 rad/s for the proposed method and 0.36 rad/s for conventional fixed-look-ahead pure pursuit. These single-run values describe the control response in the available numerical record.

It is worth noting that although MPC is close to the proposed method in path accuracy, its single-step computation time is in the millisecond range and much higher than that of the proposed controller. The proposed method adds only a small amount of adaptive computation on top of conventional pure pursuit, so it remains highly real-time and is better suited for deployment on resource-constrained agricultural robot platforms.

### 4.2. Robustness Against Localization Noise

To describe the sensitivity of the controller to prescribed positional disturbances, RMSE values are reported for positional-noise standard deviations of 0.1 m, 0.3 m, 0.5 m, and 0.8 m. The 0.5 m condition is used as the main comparison condition because the available overall performance data were obtained under this setting. The 0.8 m condition is the upper sensitivity case and is included to examine performance under a substantially increased imposed disturbance. These values are numerical sensitivity-analysis levels and are not measured greenhouse localization errors.

To quantitatively compare the sensitivity of each controller to increased positional disturbance, the relative RMSE increase from 0.1 m to 0.8 m was calculated as:

(18) $$G_{i}=\frac{{RMSE}_{i,0.8}-{RMSE}_{i,0.1}}{{RMSE}_{i,0.1}}\times 100\%$$

Here, $G_{i}$ is the relative RMSE increase in controller i, ${RMSE}_{i,0.1}$ is the RMSE of controller i at a positional-noise standard deviation of 0.1 m, and ${RMSE}_{i,0.8}$ is its RMSE at 0.8 m. A smaller $G_{i}$ indicates a smaller performance degradation over the tested disturbance range.

Based on the values in Table 2, the relative RMSE increases are 68.8% for PID, 62.9% for Stanley, 45.7% for MPC, 103.4% for conventional pure pursuit, and 36.5% for the proposed controller. The proposed controller therefore exhibits the smallest relative RMSE increase among the compared controllers, while conventional pure pursuit exhibits the largest increase. This comparison indicates a lower sensitivity of the proposed controller to the prescribed increase in positional disturbance within the tested numerical range.

The observed behavior is consistent with the design of the proposed controller. Larger innovation residuals increase the localization-noise indicator and can enlarge the look-ahead distance, thereby reducing the sensitivity of the pure-pursuit command to short-term measurement fluctuations.

### 4.3. Dynamic Characteristics of Look-Ahead Distance

**Figure 7:**
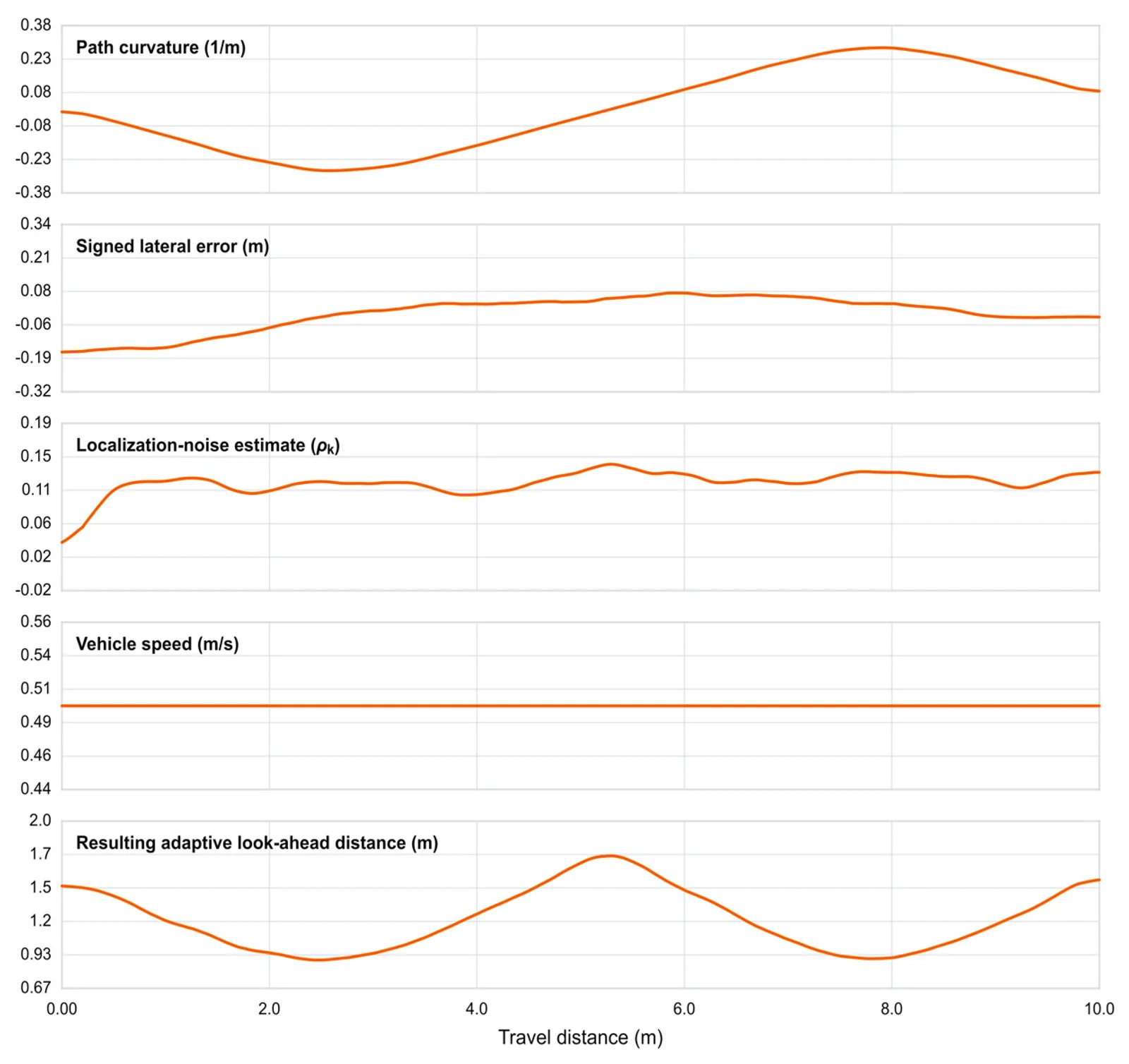
Synchronized diagnostic signals of the proposed adaptive controller on the S-shaped path.

To examine the response of the adaptive look-ahead law to changing path and feedback conditions, Figure 7 presents the synchronized variations in path curvature, signed lateral error, localization-noise estimate $\rho_{k}$, vehicle speed, and adaptive look-ahead distance $l_{d}$ along the S-shaped path.

Figure 7 presents the synchronized diagnostic signals of the proposed adaptive controller on the S-shaped path. From top to bottom, the panels show path curvature, signed lateral error, localization-noise estimate $\rho_{k}$, vehicle speed, and adaptive look-ahead distance $l_{d}$. All panels share the same travel-distance axis, enabling the variation of $l_{d}$ to be compared directly with its input signals.

The vehicle speed remains constant at 0.5 m/s; therefore, the variation in $l_{d}$ is mainly affected by path curvature, lateral error, and localization-noise feedback. The look-ahead distance decreases in the high-curvature regions near the turning portions of the S-shaped path, which improves the controller’s responsiveness to geometric changes. When the curvature magnitude decreases, $l_{d}$ gradually increases, providing smoother steering correction. Variations in the lateral error and localization-noise estimate further modulate $l_{d}$, while the look-ahead distance remains within its prescribed bounds. These results demonstrate that the proposed adaptive law can adjust the controller’s preview range according to the current path geometry and feedback condition, thereby balancing tracking responsiveness and control smoothness.

### 4.4. Ablation Study on Adaptive Control Modules

To further evaluate the role of each influencing factor in the look-ahead distance model, the retained ablation results compare the complete model with cases in which the speed, lateral-error, curvature, or noise term is removed. The corresponding results are summarized in Table 3. These results are presented as descriptive numerical comparisons, because the original trial-level data and implementation records are unavailable.

The complete model produces the lowest RMSE and angular-velocity RMS among the listed configurations. Removing the curvature term causes the largest increase in RMSE, from 0.087 m to 0.118 m, indicating that curvature information is important for tracking paths with changing geometry. Removing the localization-noise term produces the largest increase in angular-velocity RMS, from 0.28 rad/s to 0.39 rad/s, which is consistent with the role of the noise-aware mechanism in reducing sensitivity to short-term measurement fluctuations. The speed and lateral-error terms also contribute to tracking accuracy and control smoothness. These results indicate that the four factors are complementary under the specified numerical conditions, but they do not establish statistical significance or general superiority based on repeated experiments.

The available simulations were conducted at a linear speed of 0.5 m/s on predefined idealized reference paths. Therefore, the quantitative results mainly support the applicability of the proposed controller to low-speed path-tracking tasks with a known reference path. In a higher-speed scenario, the speed-related term increases the look-ahead distance and may improve steering smoothness. However, an excessively large look-ahead distance may also increase preview delay and cause corner-cutting on rapidly changing-curvature paths. Higher speed may further amplify the effects of track slip, actuator delay, chassis vibration, and unmodeled dynamic behavior. Consequently, the speed normalization parameter, weighting coefficients, look-ahead bounds, robot model, and angular-velocity constraint require further tuning and validation before application to higher-speed operation. No high-speed simulation or experimental result is available in the present study.

The proposed method is a path-tracking controller and does not include obstacle detection, obstacle prediction, collision avoidance, or online path replanning. It therefore cannot independently handle dynamic obstacles such as workers, vehicles, or temporarily blocked crop rows. In complex greenhouse environments, dynamic obstacles may require rapid path modification and may introduce additional localization occlusion and time-varying disturbances. Practical deployment would require integration with a perception system and a local planning or obstacle-avoidance module that generates an updated collision-free reference path.

In addition, the present study uses idealized reference trajectories, prescribed numerical localization disturbances, and a simplified differential-drive tracked-robot kinematic model. Detailed track-slip dynamics, terrain interaction, actuator dynamics, chassis vibration, and dynamic-obstacle behavior are not modeled. The causal SG filter may also introduce processing delay, and the controller performance may depend on the selected normalization values, weighting coefficients, and look-ahead bounds. Therefore, the available results demonstrate controller behavior under the stated numerical conditions but do not establish robustness in higher-speed operation, dynamic-obstacle environments, or real greenhouse deployment. Hardware experiments, measured greenhouse localization data, detailed dynamic modeling, and repeated-trial evaluation are required in future work.

## 5. Conclusions

This study investigated a noise-aware adaptive pure-pursuit strategy for a tracked agricultural robot using numerical simulations on idealized greenhouse-like reference paths. The controller combines EKF-based pose estimation, causal SG endpoint smoothing, and a bounded look-ahead law that considers vehicle speed, lateral error, path curvature, and an innovation-derived localization-noise indicator. The main conclusions are as follows:

1. Under the prescribed numerical localization-disturbance conditions, the EKF–SG pose-processing pipeline provides smoother pose feedback and reduces the influence of the modeled random and intermittent measurement disturbances on the subsequent control input. This result demonstrates the behavior of the proposed preprocessing scheme in the simulation model and does not constitute validation with measured greenhouse localization data.
2. The multi-factor adaptive look-ahead mechanism adjusts the look-ahead distance according to the simulated vehicle state, path geometry, tracking error, and localization-noise indicator. Under the retained 0.5 m positional-noise condition, the proposed method reports an RMSE of 0.087 m and an angular-velocity RMS of 0.28 rad/s, compared with 0.112 m and 0.36 rad/s for conventional fixed-look-ahead pure pursuit. In the retained sensitivity analysis, the relative RMSE increase from 0.1 m to 0.8 m is 36.5% for the proposed method and 103.4% for conventional pure pursuit. These values indicate lower sensitivity under the specified numerical disturbance conditions, but they are single-run descriptive results rather than statistically validated estimates.
3. The recorded computation times indicate that the proposed controller maintains a lower computational burden than the MPC comparator under the stated simulation implementation. Thus, the method retains the structural simplicity of pure pursuit and shows potential for real-time greenhouse navigation. Its suitability for practical deployment, however, requires further validation on an actual tracked robot.
4. Several limitations should be acknowledged. First, no real greenhouse field experiment or measured greenhouse localization dataset was available. The straight, circular, S-shaped, and U-shaped paths are idealized numerical reference trajectories, and the 0.1, 0.3, 0.5, and 0.8 m positional-noise levels are prescribed sensitivity-analysis conditions rather than measured greenhouse error distributions. Second, the robot is represented by a simplified differential-drive tracked-vehicle kinematic model; detailed track-slip, terrain interaction, actuator dynamics, and chassis vibration are not modeled. Third, the causal SG filter may introduce processing delay, and the controller performance may depend on the selected normalization values, weighting coefficients, look-ahead bounds, and comparator parameters. Finally, the available results are based on individual simulation records and do not provide repeated-trial uncertainty intervals or statistical significance tests. Future work should include measured greenhouse localization data, hardware experiments, detailed track-slip and dynamic modeling, parameter-sensitivity analysis, and repeated-trial statistical evaluation.

## Figures and Tables

**Table 1:** Available single-run performance values under the prescribed 0.5 m positional-noise standard deviation.

Method	RMSE (m)	Maximum Lateral Error $e_{\max}$ (m)	Angular Velocity RMS (rad/s)	Angular Velocity Rate RMS (rad/s^2^)	Average Computation Time (ms)
PID	0.156	0.381	0.46	2.13	0.11
Stanley	0.129	0.314	0.39	1.82	0.17
MPC	0.094	0.221	0.31	1.16	0.74
Pure Pursuit	0.112	0.267	0.36	1.54	0.09
Proposed	0.087	0.198	0.28	0.91	0.21

**Table 2:** Retained RMSE values under prescribed positional-noise standard deviations of 0.1 m, 0.3 m, 0.5 m, and 0.8 m.

Noise Std. (m)	PID	Stanley	MPC	Pure Pursuit	Proposed
0.1	0.112	0.097	0.081	0.089	0.074
0.3	0.131	0.108	0.087	0.101	0.081
0.5	0.156	0.129	0.094	0.112	0.087
0.8	0.189	0.158	0.118	0.181	0.101

**Table 3:** Ablation study of the proposed dynamic look-ahead distance model under different factor removal settings.

Model	RMSE (m)	Angular Velocity RMS (rad/s)
Complete model	0.087	0.28
Without speed term	0.102	0.35
Without error term	0.109	0.32
Without curvature term	0.118	0.34
Without noise term	0.096	0.39

## Data Availability

The raw data supporting the conclusions of this article will be made available by the authors upon request.

## Abbreviations

The following abbreviations are used in this manuscript:

AGV	Automated Guided Vehicle
EKF	Extended Kalman Filter
MPC	Model Predictive Control
NLOS	Non-Line-of-Sight
PID	Proportional–Integral–Derivative
RMS	Root Mean Square
RMSE	Root Mean Square Error
SG	Savitzky–Golay
